# *MT-ND5* Mutation Exhibits Highly Variable Neurological Manifestations at Low Mutant Load

**DOI:** 10.1016/j.ebiom.2018.02.010

**Published:** 2018-02-24

**Authors:** Yi Shiau Ng, Nichola Z. Lax, Paul Maddison, Charlotte L. Alston, Emma L. Blakely, Philippa D. Hepplewhite, Gillian Riordan, Surita Meldau, Patrick F. Chinnery, Germaine Pierre, Efstathia Chronopoulou, Ailian Du, Imelda Hughes, Andrew A. Morris, Smaragda Kamakari, Georgia Chrousos, Richard J. Rodenburg, Christiaan G.J. Saris, Catherine Feeney, Steven A. Hardy, Takafumi Sakakibara, Akira Sudo, Yasushi Okazaki, Kei Murayama, Helen Mundy, Michael G. Hanna, Akira Ohtake, Andrew M. Schaefer, Mike P. Champion, Doug M. Turnbull, Robert W. Taylor, Robert D.S. Pitceathly, Robert McFarland, Gráinne S. Gorman

**Affiliations:** a Wellcome Centre for Mitochondrial Research, Institute of Neuroscience, Newcastle University, Newcastle upon Tyne, UK; b Department of Neurology, Queen's Medical Centre, Nottingham, UK; c Paediatric Neurology Department, Red Cross War Memorial Children's Hospital, Cape Town, South Africa; d Division of Chemical Pathology, Faculty of Health Sciences, University of Cape Town, South Africa; e Medical Research Council Mitochondrial Biology Unit, Cambridge Biomedical Campus, Cambridge, UK; f Department of Inherited Metabolic Disease, Division of Women's and Children's Services, University Hospitals Bristol NHS Foundation Trust, Bristol, UK; g Tongren Hospital, Shanghai Jiaotong University School of Medicine, Shanghai, China; h Royal Manchester Children's Hospital, Central Manchester University Hospitals NHS Foundation Trust, UK; i Institute of Human Development, University of Manchester, Manchester M13 9WL, UK; j Ophthalmic Genetics Unit, OMMA, Institute of Ophthalmology, Athens, Greece; k Pediatric Ophthalmology Department, MITERA Children's Hospital, Athens, Greece; l Radboud Center for Mitochondrial Medicine, Department of Pediatrics, Radboud University Medical Center, Nijmegen, The Netherlands; m Department of Neurology, Radboud University Medical Center, Nijmegen, The Netherlands; n Department of Pediatrics, Nara Medical University Hospital, Nara 634-8522, Japan; o Department of Pediatrics, Sapporo City General Hospital, Sapporo 060-8604, Japan; p Diagnostics and Therapeutics of Intractable Diseases, Intractable Disease Research Center, Graduate School of Medicine, Juntendo University, Tokyo 113-8421, Japan; q Department of Metabolism, Chiba Children's Hospital, Chiba 266-0007, Japan; r Evelina London Children's Hospital, Guy's & St Thomas' NHS Foundation Trust, London, UK; s MRC Centre for Neuromuscular Diseases, UCL Institute of Neurology and National Hospital for Neurology and Neurosurgery, London, UK; t Department of Pediatrics, Faculty of Medicine, Saitama Medical University, Saitama 350-0495, Japan; u National Health Laboratory Service, Cape Town, South Africa; v Central Manchester University Hospitals NHS Foundation Trust, Manchester Academic Health Science Centre, Manchester M13 9WL, UK

**Keywords:** Mitochondrial encephalomyopathy, Lactic acidosis and stroke-like episodes (MELAS), Leigh syndrome (LS), Mitochondrial DNA, Heteroplasmy, Neuropathology

## Abstract

Mutations in the m.13094T>C *MT-ND5* gene have been previously described in three cases of Leigh Syndrome (LS). In this retrospective, international cohort study we identified 20 clinically affected individuals (13 families) and four asymptomatic carriers. Ten patients were deceased at the time of analysis (median age of death was 10 years (range: 5·4 months−37 years, IQR = 17·9 years). Nine patients manifested with LS, one with mitochondrial encephalomyopathy, lactic acidosis and stroke-like episodes (MELAS), and one with Leber hereditary optic neuropathy. The remaining nine patients presented with either overlapping syndromes or isolated neurological symptoms. Mitochondrial respiratory chain activity analysis was normal in five out of ten muscle biopsies. We confirmed maternal inheritance in six families, and demonstrated marked variability in tissue segregation, and phenotypic expression at relatively low blood mutant loads. Neuropathological studies of two patients manifesting with LS/MELAS showed prominent capillary proliferation, microvacuolation and severe neuronal cell loss in the brainstem and cerebellum, with conspicuous absence of basal ganglia involvement. These findings suggest that whole mtDNA genome sequencing should be considered in patients with suspected mitochondrial disease presenting with complex neurological manifestations, which would identify over 300 known pathogenic variants including the m.13094T>C.

## Highlights

• The m.13094T>C mutation in *MT-ND5* is associated with severe, variable neurological manifestations.
• Whole mtDNA genome sequencing should be considered in patients with undiagnosed complex neurological disorders.
• Biochemical analysis of muscle maybe normal in more than half of all cases.

The m.13094T>C mutation in *MT-ND5* was considered a rare cause of mitochondrial disease that had been previously reported in association with Leigh Syndrome only. This study revealed that the m.13094T>C mutation is associated with severe, variable neurological features, with a more extensive phenotypic spectrum of disease, frequently manifesting at lower mutant heteroplasmy levels compared to many other primary mitochondrial DNA mutations. The work described here proposes that an unexplained central nervous system disorder should raise clinical suspicion of a mitochondrial disorder, and full mtDNA genome sequencing should be considered even with normal muscle biopsy findings.

## 1 Introduction

Defects in oxidative phosphorylation (OXPHOS) are an important cause of human morbidity and mortality, with complex I (NADH-ubiquinone oxidoreductase) deficiency recognized as the most commonly observed OXPHOS defect (Rodenburg, 2016). Complex I (NADH: ubiquinone oxidoreductase) is the largest component of the oxidative phosphorylation system (OXPHOS) composed of 45 subunits that, in supercomplex formation with respiratory chain complexes III and IV, drives the generation of a transmembrane protein gradient powering adenosine triphosphate (ATP) synthesis. Complex I requires 14 evolutionary conserved core subunits for its catalytic function: seven mtDNA-encoded NADH-dehydrogenase (ND) core subunits (ND1-6, ND4L) and seven nuclear DNA (nDNA) encoded subunits (NDUFV1, NDUFV2, NDUFS1, NDUFS2, NDUFS3, NDUFS7 and NDUFS8), in addition to 31 supernumerary subunits, whose exact roles are yet to be fully defined (Zhu et al., 2016). The *MT-ND5* gene of complex I, appears to be a mutational ‘hot spot’ (Bannwarth et al., 2013) and linked to a variety of clinical phenotypes ranging from single organ involvement, such as isolated exercise intolerance (Downham et al., 2008) or Leber hereditary optic neuropathy (LHON) (Howell et al., 1993), to multisystem disease manifesting as renal failure and myopathy (Alston et al., 2010), Leigh syndrome (LS) (Blok et al., 2007; Kirby et al., 2003), mitochondrial encephalomyopathy, lactic acidosis and stroke-like episodes (MELAS) (Shanske et al., 2008; Liolitsa et al., 2003), or as a combination of overlapping syndromes including LS/MELAS (Blok et al., 2007; Crimi et al., 2003), MELAS/myoclonic epilepsy and ragged red fibers (MERRF) (Naini et al., 2005), LHON/MELAS (Pulkes et al., 1999) and LS/MELAS/LHON (Liolitsa et al., 2003). The limited understanding of the natural history of disease caused by such mutations presents significant challenges in clinical practice, particularly in relation to pre-symptomatic genetic testing of at-risk family relatives.

The m.13094T>C mutation, in the *MT-ND5* gene, is considered a rare pathogenic variant that has been previously reported in association with LS but the full phenotypic spectrum has remained poorly understood (Valente et al., 2009; Ching et al., 2013). We present the clinical, radiological, and histopathological data of 24 subjects who harbor the m.13094T>C mutation. We have also studied the neuropathological changes in two patients with LS/MELAS overlap syndrome, to fully elucidate the spectrum of m.13094T>C-related mitochondrial disease and to offer guidance on management and genetic counseling.

## 2 Materials and Methods

### 2.1 Study Design and Patients

This retrospective, international cohort study was done at the NHS Highly Specialised Service-funded Mitochondrial Diagnostic Centers in Newcastle upon Tyne and London, UK. These included patients referred from four other countries: China, South Africa, Greece and Japan. Eligible participants were genetically confirmed to harbor the m.13094T>C mutation (p.Val253Ala) in *MT-ND5* over a 17 year period (January 2000–October 2017); their maternal family members were traced and examined whenever possible.

This study was approved and performed under the ethical guidelines issued by our institution for clinical studies, and complied with the declaration of Helsinki.

### 2.2 Clinical Presentation and Phenotypic Evaluation

Each patient was assessed by a specialist (pediatrician, adult neurologist and/or ophthalmologist) at each center, and their medical records were comprehensively reviewed. The clinical presentation of individual patients and their family pedigree are provided in Supplemental data and Supplemental Fig. 1. Where possible, common clinical syndromes described in mitochondrial disease were assigned (and agreed by all authors).

### 2.3 Histopathological and Biochemical Studies

Standard histological (hematoxylin and eosin (H&E) and modified Gomori Trichome stains), histochemical (cytochrome *c* oxidase (COX), succinate dehydrogenase (SDH), and sequential COX–SDH and biochemical assessments of muscle tissue were performed as described elsewhere (Supplemental Table 1). Fresh tissue was fixed in 2.5% glutaraldehyde buffered to pH 7.4 with phosphate buffer and post-fixed in OsO4 and embedded in Epon LX-112. Ultra-thin sections were examined in an electron microscope (JEOL⁃1500, Japan) for Patient 5.

### 2.4 Molecular Genetics

Extraction of total DNA was performed as per standard procedure. The whole mitochondrial genome was sequenced, and pyrosequencing assay was used (Blakely et al., 2013) to screen for and quantify the m.13094T>C mutation (GenBank Accession number: NC_012920.1) for 13 patients (Patients 1, 1.1, 1.2, 2, 2.1, 2.2, 2.4, 3, 3.1, 4, 6, 8 and 8.1). The assay could reliably detect a level of >3% of mutant mtDNA. Restriction fragment length polymorphism (RFLP) analysis was performed to quantify the m.13094T>C heteroplasmy level in Patients 5, 12 and 13, with the sensitivity of ~5% (Valente et al., 2009). The mutant heteroplasmy level was quantified using next-generation sequencing (NGS) for Patients 7, 7.1, 7.2, 9 and 10 (sensitivity > 10%).

### 2.5 Neuropathological Studies

Neuropathological investigations were performed on postmortem brain tissues from two patients (patients 1.2 and 8). Cresyl fast violet (CFV), H&E and Luxol fast blue with H&E counterstain were used on formalin-fixed paraffin-embedded tissues to determine neuronal population density and degree of myelination. Immunohistochemistry to determine the expression levels of mitochondrial respiratory chain subunits, including complex I subunit NADH: ubiquinone oxidoreductase subunit B8 (NDUFB8) and complex II subunit succinate dehydrogenase subunit A (SDHA), were performed on 5 μm thick sections as previously described (Lax et al., 2012).

### 2.6 Statistical Analysis

Non-parametric, continuous data were presented as median (range and inter-quartile range (IQR)). Correlation of mtDNA heteroplasmy level of different tissues and age of disease onset was examined using Spearman rank correlation test. Statistical significance was determined at *p* < 0.05. Data were managed and analyzed with IBM SPSS for Windows version 22.

## 3 Results

### 3.1 Patient Cohorts

Clinical features of 24 individuals from 13 families are summarised in Table 1. The median age of disease onset was 5.5 years (*n* = 20, range: 6 weeks to 34 years, IQR = 13 years; the age of onset not known for two patients). Four individuals were clinically unaffected; they were ascertained pre-symptomatically due to diagnosis in other family members. Ten patients (42%) were deceased, and the median age of death was 10 years (range: 5.4 months to 37 years, IQR = 17.9 years).

**Table 1:** Syndromic classification and clinical features of individuals with the m.13094T>C mutation (*n* = 24).

Pedigree patient	Relation	AO/AL*	Syndrome	Clinical features													Heteroplasmy (%)		
				Sz	SLEs	Cog	Str	ON	Pt	Ap	DD	At	PN	LA	Cardiac	Other	B	U	Mu
1 (F)	Proband	17/37*	LHON/MELAS	+	+	+	−	+	−	−	−	+	+	−	−	−	0	55	n.d.
1·1 (M)	Offspring	2 mths/9 mths*	LS	−	−	n.k.	−	−	−	+	+	−	n.k.	+	−	−	n.d	n.d.	65
1·2 (F)	Offspring	10/14*	MELAS/LS	+	+	+	+	+	+	−	−	+	+	+	−	−	46	67	90
2 (F)	Proband	12/30	SCA/MELAS	+	+	+	−	−	+	−	−	+	+	−	−	−	9	40	35
2·1 (F)	Mother	n.k./49	Ataxia	−	−	−	−	−	−	−	−	+	−	−	−	−	3	22	n.d.
2·2 (F)	Maternal grandaunt	−/74	None	−	−	−	−	−	−	−	−	+	−	−	−	Ischaemic stroke, tremor	0	n.d.	n.d.
2·3 (M)	Maternal cousin	4 mths/1.5*	LS	n.k.	n.k.	n.k.	n.k.	n.k.	n.k.	+	n.k.	n.k.	n.k.	n.k.	n.k.	−	n.d.	n.d.	n.d.
2·4 (M)	Maternal cousin	5/43	LS-like/SCA	−	−	−	+	+	−	−	−	+	−	−	−	−	29	n.d.	n.d.
3 (M)	Proband	6 wks/5.4 mths*	LS	+	−	n.k.	+	−	+	+	+	−	−	+	−	−	76	81	80
3·1 (F)	Mother	−/20s	Unaffected	−	−	−	−	−	−	−	−	−	−	−	−	−	19	45	n.d.
4 (M)	Proband	4/6*	LS	−	−	−	+	−	+	+	+	+	−	Raised CSF	HCM	Spasticity	n.d.	n.d.	71
4·1 (F)	Mother	−/n.k.	Unaffected	−	−	−	−	−	−	−	−	−	−	−	n.k.	−	4	9	n.d.
5 (F)	Proband	13/14*	MELAS/LS	+	+	+	+	−	+	+	−	−	−	+	−	Myoclonus	n.d.	n.d.	28
6 (M)	Proband	2.25/3*	LS	−	−	n.k.	+	−	+	+	+	+	−	−	Brady	−	45	n.d.	58
7 (M)	Proband	7/21	LHON	−	−	−	−	+	−	−	−	−	−	−	−	−	26	n.d.	n.d.
7·1 (M)	Brother	6/20	Subclinical ON	−	−	−	−	+	−	−	−	−	−	−	−	−	35	n.d.	n.d.
7·2 (F)	Mother	−/47	Unaffected	−	−	−	−	−	−	−	−	−	−	−	−	−	7	n.d.	n.d.
8 (F)	Proband	34/35*	MELAS/LS	+	+	+	+	−	+	−	−	+	−	−	PFO	Myoclonus	n.d.	n.d.	83
8·1 (F)	Mother	−/70s	Unaffected	−	−	−	−	−	−	−	−	−	−	−	−	−	0	6	n.d.
9 (F)	Proband	27/34	MELAS	+	+	+	−	–	–	−	−	+	–	+	–	–	5	n.d.	80
10 (M)	Proband	22/24	LS	−	−	−	+	–	+	+	−	+	−	−	LVH	NIV B/L INO	38	n.d.	61
11 (M)	Proband	2/30	LS	–	–	–	+	+	–	+	−	+	–	Raised CSF	–	PEG, dystonia, trachy, facial weakness	n.d.	n.d.	n.d.
12 (M)	Proband	1/4	LS	−	−	−	+	−	+	−	+	−	−	+	−	−	n.d.	n.d.	41 (Fib)
13 (M)	Proband	1/14*	LS	+	−	+	+	−	+	+	+	n.k.	−	+	−	Trachy, PEG	49	n.d.	52
			Total	8/23	6/23	7/20	11/23	6/23	10/23	9/24	6/23	12/22	3/22	9/23	4/22				

* = deceased, AO = age of onset, AL = age of last follow up or death, Ap = apnoea, At = ataxia, B = blood, B/L INO = bilateral inter-nuclear ophthalmoplegia, Brady = bradycardia, Cog = cognitive impairment, Dev delay = developmental delay (including motor and speech), F = female, Fib = fibrolast, HCM = hypertrophic cardiomyopathy, HTN = hypertension, LA = lactic acidosis, LS = Leigh syndrome, LVH = left ventricular hypertrophy, M = male, MELAS = mitochondrial encephalomyopathy, lactic acidosis and stroke-like episodes, mths = months, Mu = muscle, n.d. = not done, NIV = non-invasive ventilation, n.k. = not known, ON = optic neuropathy, PEG = percutaneous endoscopic gastrostomy, PFO = patent foramen ovale, PN = peripheral neuropathy, Psy = neuropsychiatric symptoms such as severe depression, anxiety or personality change, Pt = ptosis, RF = respiratory failure, SCA = spinocerebellar ataxia, SLE = stroke-like episodes, Str = strabismus, Sz = seizures, trachy = tracheostomy, U = urine, yrs. = years.

Eleven patients presented with distinctive clinical syndromes previously described in mitochondrial disease. However, the remaining patients (*n* = 9) manifested with an isolated neurological symptom or overlapping syndromes, of which central nervous system was most commonly affected (Table 1). Centrally mediated respiratory failure or apnoea was documented in nine patients (38%) with LS, including an adult patient who presented at the age of 22 years (Patient 10). Refractory focal onset seizures with or without evolution to bilateral convulsions occurred in eight patients (35%).

### 3.2 Radiological Imaging

**Fig. 1:**
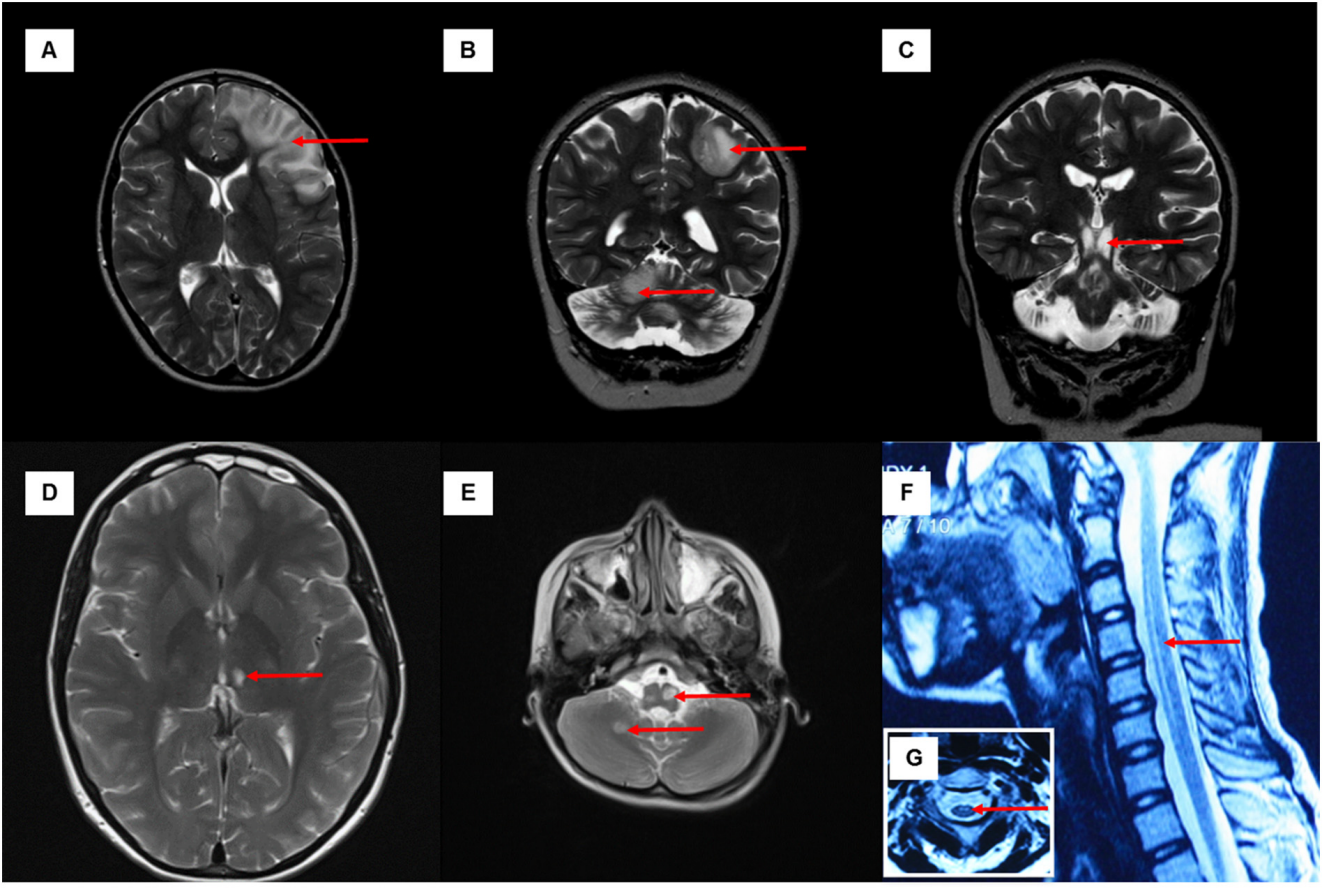
Cranial and spine MRI. Axial T2-weighted view of Patient 1·2 showed hyperintensities involving the cortical and subcortical areas of left frontal lobe when she presented with the first stroke-like episode (A); the cranial MRI performed during the subsequent stroke-like episode showed hyperintensities involving the left parietal lobe and right cerebellar cortex, suggestive of cross cerebellar diaschisis (B) and extensive signal abnormalities in the brainstem (C, coronal view). Axial T2-weighted view of Patient 2 showed an isolated, left thalamic lesion (D). Axial T2 view (E) showed a discrete signal abnormality in the right cerebellum, and asymmetrical hyperintensities in the medulla in Patient 4. The sagittal T2 view (F) showed an anterior, long hyperintensity in the cervical cord spanning C2-6 levels in Patient 5, corroborated with the signal abnormalities shown in the axial view (G).

Cranial magnetic resonance imaging (MRI) was available for analysis in 17 patients (Table 2, Fig. 1 and Supplemental Fig. 2). The most common T2/FLuid Attenuation Inversion Recovery (FLAIR)-signal abnormalities were present in the midbrain (*n* = 12) and thalamus (n = 9: bilateral, symmetrical changes; *n* = 3: unilateral) followed by pons (*n* = 10) and medulla (n = 9). Brainstem changes in two patients (Patients 6 and 8), initially was initially misdiagnosed as a low-grade glioma, although gadolinium-enhancement was absent. Subacute cortical and subcortical signal changes were identified in six patients that were consistent with stroke-like lesions. Two patients had multiple cortical lesions including lesions within the cerebellar hemispheres (Patients 1.2 and 5), suggestive of cross cerebellar diaschisis. Cervical cord lesions were also identified in six patients.

**Table 2:** Cranial MRI changes associated with the m.13094T>C mutation (*n* = 17).

Patient no	Age at scan (years)	Brain imaging anomalies										Fluctuating/relapsing-remitting imaging changes
		Cerebral cortex stroke-like lesion	Basal ganglia	Thalamus	Cerebellum			Brainstem			Cervical cord	
					Dentate nuclei	Cortex	Atrophy	Midbrain	Pons	Medulla		
1	36	P, T (R)	−	U/L (R)	−	−	+	−	−	−	−	Yes
1.1	8 mths	−	−	B/L	−	−	−	+	+	+	C1–2	n.k.
1.2	13; 14	P, O, H (L)	−	B/L	+	+	+	+	+	+	C1	Yes
2	26; 30	O (R)	−	U/L (L)	−	−	−	−	−	−	−	Yes
2.2	66	Δ	−	−	−	−	−	−	−	−	−	No
2.4	31	−	−	B/L	+	+	+	+	+	+	−	n.k.
3	3 mths	−	−	B/L	−	−	−	+	+	+	+*	n.k.
4	6	−	−	−	+	−	−	+	−	+	−	n.k.
5	13; 14	F (B/L), P (B/L)	−	B/L	−	+	+	+	+	−	C2–6	Yes
6	2.25	−	−	B/L	−	−	−	+	+	+	−	No
7	7	−	−	−	−	−	−	−	−	−	−	n.k.
8	33	O (R)	−	B/L	−	−	−	+	−	−	−	Yes
9	34	O (B/L), P,T,F (L)	–	−	−	−	−	−	−	−	n.k.	Yes
10	24	−	−	−	−	−	−	+	+	+	−	n.k.
11	22, 23	−	+	U/L	−	−	+	+	+	+	+ (upper)*	Yes
12	1 yr 4 mths	−	−	B/L	−	−	−	+	+	+	n.k.	Yes
13	10	−	+	B/L	−	−	−	+	+	−	−	No
	Total	6/17	2/17	12/17	3/17	3/17	5/17	12/17	10/17	9/17	5/15	8/11

All cortical lesions (including cerebellar cortex) exhibited restricted diffusion. B/L = bilateral, CR = corona radiata, F = frontal lobe, H = hippocampus, L = left, n.k. = not known, O = occipital lobe, P = parietal lobe, R = right, T = temporal lobe, U/L = unilateral, Δ = Patient 2·2 had a history of clinically and radiologically defined lacunar stroke (internal capsule and corona radiate), * = the extent of cervical lesion had not been clearly defined.

Signal abnormalities within the basal ganglia, classically seen in LS, were only identified in two patients (Patients 11 and 13).

### 3.3 Histopathological and Biochemical Analyses

Histopathological and histochemical evaluation was normal in most patients except minor changes detected in the skeletal muscle of three patients: minor ragged red fibers (n = 1), lipid droplets and abnormal mitochondrial ultrastructure (n = 1) and occasional angular atrophic fibers (n = 1). Mitochondrial respiratory chain activity was measured in skeletal muscle tissue from 10 patients. One patient demonstrated isolated complex I deficiency, one patient with combined complex I and III deficiencies, two patients with combined complex I and IV deficiencies and one patient had low complex I and II activity in postmortem tissue, which was caused by significant delay in tissue handling. The remaining six cases demonstrated normal respiratory chain activity (Supplemental Table 1).

### 3.4 Molecular Genetics

Thirteen probands were identified to harbor the m.13094T>C mutation through whole mtDNA sequencing after screening negative for common mtDNA point mutations including m.3243A>G, m.8344A>G, *MTATP6*, and *MTATP8* genes. The mutation was identified in nine individuals by direct sequencing of the mtDNA point mutation through pedigree and segregation analysis. Maternal inheritance of the m.13094T>C mutation was confirmed in six family pedigrees. The m.13094T>C mutation was not detected in the blood of Patient 1 (measured at age 37 years) with a severe, adult-onset MELAS phenotype although was detected at 51% in urine. Patient 2.2 was presumed an obligate carrier even though the m.13094T>C mutation was not detectable in blood (measured at age 74 years). There was no tissue sample available for testing in Patient 2.3. The asymptomatic mother of patient 3 was demonstrated to harbor the m.13094T>C mutation at mtDNA heteroplasmy levels of 19%, 27% and 45% in blood, muscle, and urine respectively (measured at age 24 years). The quantification of mutant mtDNA heteroplasmy level was not performed in two patients due to no access to tissue samples (Patient 2.3) and only Sanger sequencing was performed in Patient 11 (the mutant load in muscle appeared higher than blood; sensitivity > 25%).

**Fig. 2:**
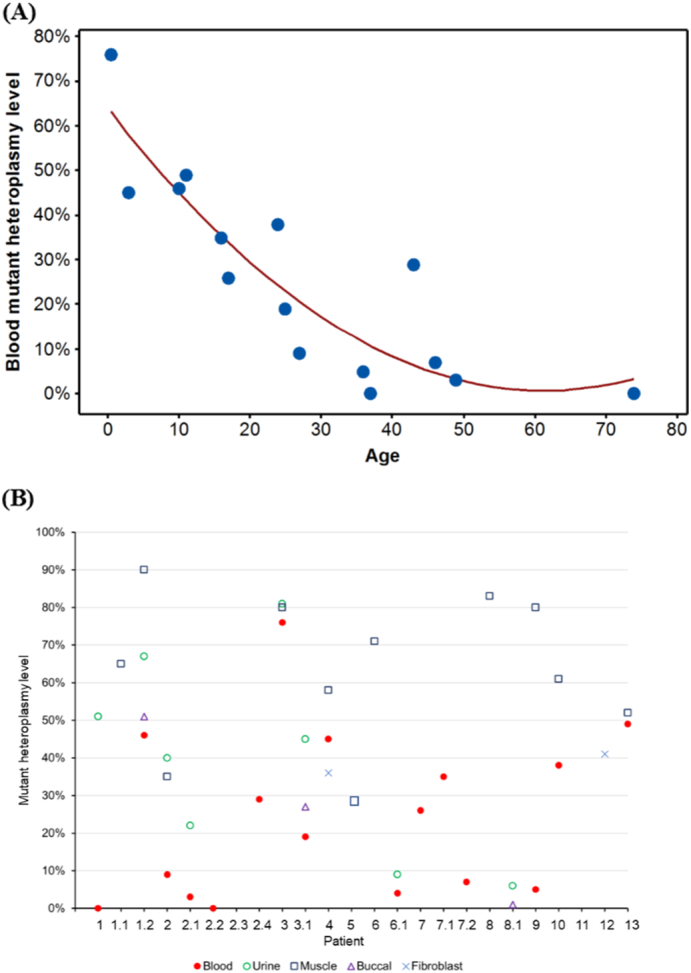
Heteroplasmy levels of the m.13094T>C mutation. (A) A negative correlation between age and blood mutant heteroplasmy level. (B) Distribution of mutant heteroplasmy levels for individual patient. The numbering of X-axis corresponds to the patient number of Table 1.

There was a statistically significant negative correlation between blood mtDNA heteroplasmy level and age (Spearman rho = −0.883, *p* < 0.001; Fig. 2A). Among the patients with mutant mtDNA load quantified in more than one tissue (*n* = 12), the mutant heteroplasmy levels in muscle (65% ± 20%; *n* = 10) and/or urine (40% ± 27%; *n* = 8) were consistently higher than blood (24% ± 22%; *n* = 16) (Fig. 2B). The discrepancy between muscle and blood mtDNA heteroplasmy ranged from 3 to 75% in seven patients; the discrepancy between urine and blood mtDNA heteroplasmy levels ranged from 5 to 51% in seven patients. Whole exome sequencing (methods described elsewhere (Neveling et al., 2013)) was performed to investigate the cause of optic neuropathy in Patient 7. This excluded other known causative genes (including *OPA1)*.

The m.13094T>C mutation load of a number of post-mortem tissues was analyzed from Patients 1.2 and 13 exemplifying the varied tissue segregation that is characteristic of this mtDNA mutation (Table 3). The m.13094T>C mutation load was specifically determined in brain homogenate samples from patients 1.2 and 8 demonstrating lower mutant load within neuronal tissues (median = 61%, range: 47–69%) compared to other tissues despite both manifesting with extensive neurological sequelae (Table 3).

**Table 3:** The m.13094T>C heteroplasmy levels in different post-mortem tissues.

	Patient 1.2	Patient 8	Patient 13
Syndrome	MELAS/LS	MELAS/LS	LS
Age of death	14	35	14
Post-mortem tissues			
Heart	75%	–	64%
Adrenal Gland	96%	–	–
Liver	83%	–	23%
Kidney	83%	–	56%
Bladder	90%	–	
Skeletal muscle	88%	–	52%
Intestine	69%	–	24%
Lung	–	–	28%
Frontal lobe	69%	47%	–
Hippocampus	67%	52%	–
Cerebellum	64%	58%	–

Muscle (Spearman rho = 0.192, *p* = 0.62) and urine (Spearman rho = −0.80, *p* = 0.20) mutant mtDNA heteroplasmy levels did not correlate with the age of disease onset. There was no significant difference in mean mutant heteroplasmy level in different syndromic categories (*p* = 0.122).

### 3.5 Neuropathological Findings

**Fig. 3:**
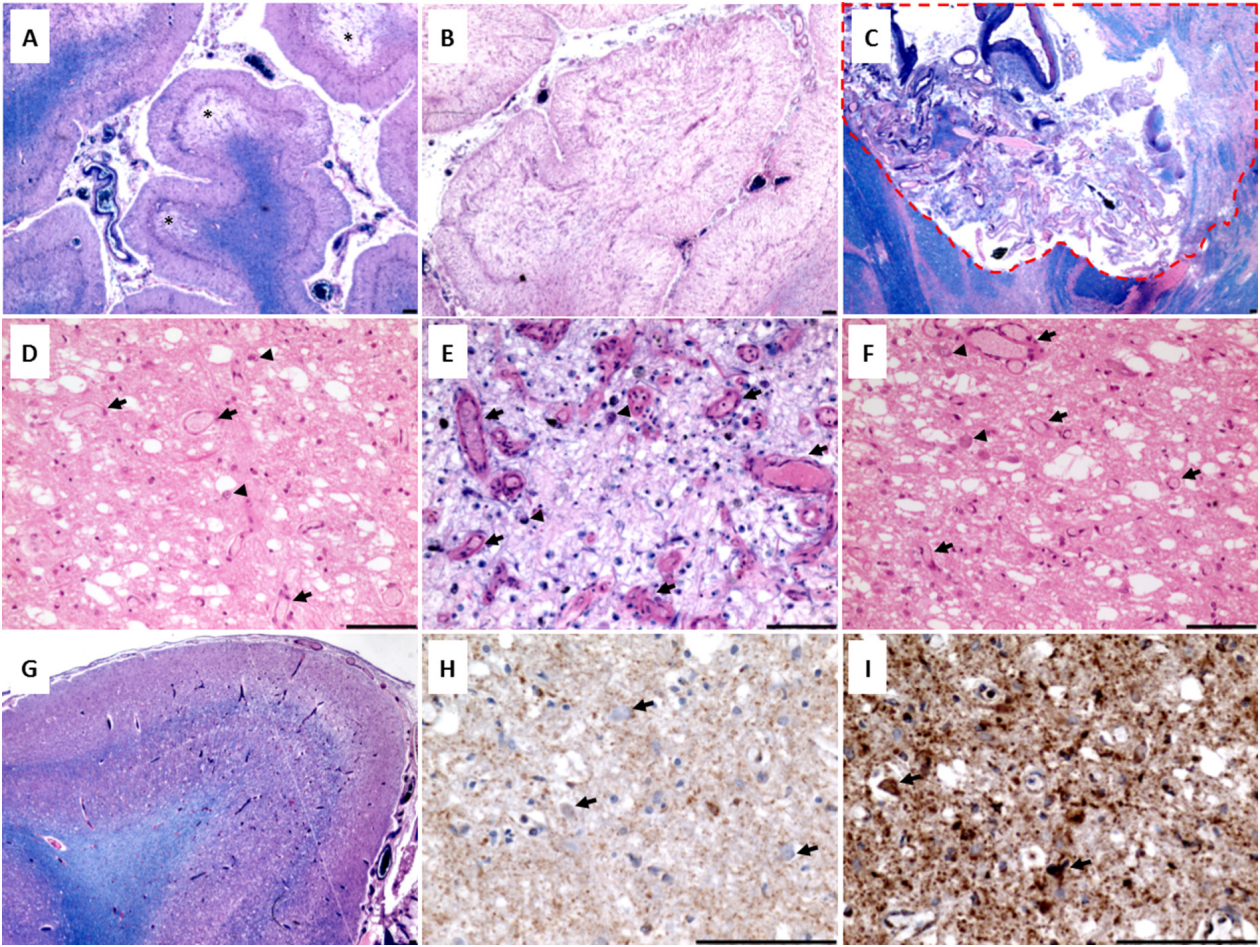
Neuropathological features of Patient 1.2 and 8. Patient 1.2: The posterior cerebellar cortex is affected by multiple necrotic lesions (demarcated by *) demonstrating atrophy of the molecular layer, Purkinje cell dropout and granule cell loss (A; LFB H&E) and total necrosis of the cortex and underlying white matter (B; LFB H&E). The basis pontis demonstrates a devastating lesion with total neuronal cell loss (C; red dashed line; LFB H&E). Scale bar = 100 μm. Patient 8: The lower midbrain shows devastated inferior colliculus with prominent capillary proliferation (arrows), microvacuolation and severe neuronal cell loss (D; H&E). The thalamus (E; LFB H&E) and subthalamic nucleus (F; H&E) are devastated featuring prominent capillary proliferation (arrows), microvacuolation, severe neuronal cell loss and morphologically normal neurons scattered throughout (arrowhead). The occipital lobes reveal microvacuolation and laminar necrosis of the cortical layers within Broadmann area 17 (G; LFB H&E) with the underlying white matter demonstrating myelin pallor relative to otherwise preserved myelin. The cortex features microvacuolation, severe neuronal cell loss and capillary proliferation. Surviving neurons within the inferior colliculus lack complex I subunit expression (H; NDUFB8 IHC, arrows) while mitochondrial mass is high (I; SDHA IHC, arrows).

The major neuropathological findings for patients 1.2 and 8 are summarised in Supplemental Table 2). In patient 1.2, posterior cerebellar cortex demonstrated multiple areas of necrotic lesions ranging from atrophy of the molecular layer, Purkinje cell dropout and granule cell loss (Fig. 3A), to total necrosis of the cerebellar cortex that also affected the underlying white matter (Fig. 3B). Concerning topographical distribution, lesions were most severe in the brainstem nuclei (Fig. 3C and D), the thalamic and subthalamic nuclei (Fig. 3E and F) and primary visual cortex (Brodmann area 17) (Fig. 3G). At a microscopic level, changes such as prominent capillary proliferation, microvacuolation and severe neuronal loss were frequently observed in fixed cerebral and cerebellar hemispheres, and right brainstem compatible with LS. Downregulation of complex I subunit (NDUFB8) was evident in the in the pons and cerebellar cortex of patient 1.2, and lesioned thalamus and occipital cortex of patient 8 (Supplemental Fig. 3).

## 4 Discussion

In this study, we have identified marked clinical heterogeneity with a continuous spectrum of overlapping symptoms associated with the m.13094T>C mutation. However within that clinical heterogeneity, at certain points in the course of disease, several distinct clinical syndromes associated with mutations in the *MT-ND5* gene were clearly discernible: early onset LS (38%), late childhood/early adulthood-onset LS/MELAS overlap syndrome (13%) and LHON (4%) (not previously recognized in association with the m.13094T>C mutation). LS frequently manifested with a typical illness trajectory including hypotonia, cranial nerve palsies, cerebellar ataxia, and developmental regression; with relapses in clinical status often triggered by inter-current illness. Brainstem dysfunction (manifesting as centrally-mediated respiratory failure and lability in blood pressure), commonly occurred as a pre-terminal event. Patients with the LS/MELAS overlap syndrome typically presented with refractory focal seizures and stroke-like lesions (as classically seen in MELAS). Additional features of brainstem dysfunction, including acute-onset ptosis, a complex eye movement disorder and worsening ataxia (as classically seen in LS) were not infrequent. Neuropsychiatric symptoms such as excessive anxiety, low mood, and hypersomnolence, in addition to cognitive impairment were features accompanying stroke-like episodes. A progressive gait disorder with truncal ataxia was a prominent clinical feature in three adult patients (13%), and additional neurological features such as strabismus (Patient 2.4), axonal neuropathy and stroke-like episode (Patient 2) were identified. Interestingly, optic neuropathy was the first clinical manifestation in four patients (Patients 1, 2.4, 7 and 7.1); however, additional severe central nervous system (CNS) involvement evolved in two patients (Patients 1 and 2.4). These findings highlight that as the disease progressed, discrete syndromes were no longer discernible. Indeed, the heterogeneity of the neurological manifestations in the m.13094T>C mutation appears similar to that observed in other mtDNA encoded complex I gene mutations.

The most common T2/FLAIR signal abnormalities on cranial MRI were localized to the brainstem (71%), thalamus (71%), cerebral cortex (35%) and cervical cord (31%) and medial thalamic changes (with restricted diffusion) were identified in seven out of eight patients presenting with refractory epilepsy. Such findings suggest prolonged seizure activity (focal status epilepticus) as the underlying pathophysiological mechanism of the radiological (and clinical) changes observed (Katramados et al., 2009). MRI abnormalities in cortical and/or subcortical areas, cerebellar hemispheres (crossed cerebellar diaschisis, which refers to cerebellar hypometabolism is ascribed to functional disconnection of the contralateral hemisphere from the cerebral cortex) (Tien and Ashdown, 1992) corpus callosum, thalami and basal ganglia have been increasingly reported in cases of isolated seizures, seizure clusters, and status epilepticus (Katramados et al., 2009). Moreover, the development of cognitive dysfunction in these patients may be explained by the observed involvement of the cerebello-thalamo-cortical pathway (white matter tracts connecting the cerebellar cortex to the contralateral various cerebral cortices, passing through the superior cerebellar peduncle, red nuclear and the thalamus) (Palesi et al., 2015). T2/FLAIR signal abnormality involving the brainstem extending into the upper cervical cord was identified in four patients. Interestingly, Patient 5 had quite extensive T2/FLAIR signal changes spanning from C2 to C6, mimicking a demyelinating disorder but CSF restricted oligoclonal bands and anti-aquaporin 4 antibodies both tested negative. Whilst MR imaging findings are consistent with previously documented changes in other complex I deficient patients, it is striking that only two patients with the m.13094T>C mutation had evidence of striatal lesions (Valente et al., 2009) compared to 90% of complex I cases reported by Lebre et al. (2011)) These findings are corroborated by relative preservation of the striatum and the presence of devastating destruction of the thalami and brainstem with marked capillary proliferation on autopsy examination. Whilst symmetrical signal abnormalities in basal ganglia are the most common radiological finding in patients affected by LS, lesions may merely be confined to the brainstem (Leigh, 1951; Rahman et al., 1996), as was observed in all our cases. This again serves as a reminder that the diagnosis of LS (including due to *MT-ND5* mutations) should be considered even in the absence of symmetrical basal ganglia lesions.

The radiological appearances of patients with LS harboring the m.13094T>C mutation bear similarities to mitochondrial diseases caused by autosomal recessive tRNA synthetase mutations, such as leukoencephalopathy with thalamus and brainstem involvement and high lactate (LTBL) caused by *EARS2* mutations and leukoencephalopathy with brainstem and spinal cord involvement and lactate elevation (LBSL) caused by *DARS2* mutations (Diodato et al., 2014). In two of our cases, the radiological diagnosis was thought to be a space- occupying lesion at presentation. However, the ‘relapsing-remitting’ course of neurological deficits associated with m.13094T>C*-*related mitochondrial disease, coupled with the evolution of signal abnormalities on interval brain imaging and demonstration of abnormal signals in different regions of structurally normal brain on T1 images (but abnormal cerebral metabolism on MR spectroscopy), should aid clinicians to discern between the two conditions.

The clinical features of these patients may also overlap at various stages of the disease with other more common forms of mitochondrial disease, such as LHON*, OPA1*, and *POLG*-related disease, and may mimic other forms of hereditary ataxia. In addition, m.13094T>C-related mitochondrial disease may mimic inflammatory CNS disorders such as acute disseminated encephalomyelitis (ADEM), multiple sclerosis (MS) and neuromyelitis optica (Patients 5, 6 and 7). Indeed, the link of an MS-like illness and common LHON mutations is well recognized in Harding disease, where imaging appearances are frequently indistinguishable from MS (Matthews et al., 2014). More recently, the coincidental occurrence of an MS-like disorder and *OPA1* mutations in three unrelated patients has also been reported (Yu-Wai-Man et al., 2016).

We observed that the histochemical changes such as ragged red and COX deficient fibers, and biochemical analysis of mitochondrial respiratory chain activity in muscle tissue, was normal in 60% of the cases presented here, similar to other point mutations in *MT-ND5* (Kirby et al., 2003; Liolitsa et al., 2003; Crimi et al., 2003). In addition, the quadruple immunohistochemistry technique could not detect the reduction of complex I subunit in muscle tissue in one of our patients (Ahmed et al., 2017), in accordance with the findings of preferential CNS manifestations in association with this mutation. Interestingly, the threshold level of mutant mitochondrial load necessary to cause symptoms in these cases appears to be much lower than in mitochondrial tRNA gene-related disorders associated with severe clinical phenotypes. However, our findings corroborate previous findings in transmitochondrial cybrid models of the m.13094T>C mutation (Valente et al., 2009). Furthermore, severe clinical phenotypes associated with a relatively low mutant load have also been observed in other common point mutations in the *MT-ND5* gene, including the m.13513G > A mutation, where a mutant load of <50% in muscle has been reported in association with LS (Kirby et al., 2003) and MELAS (Shanske et al., 2008). Preliminary pedigree analysis did not *initially* prove informative; however, extensive segregation testing confirmed this to be a maternally inherited genetic disorder. In addition, our findings highlight the importance of recognizing that mtDNA disease can only be definitively excluded by performing whole mitochondrial genome sequencing in post-mitotic tissues, such as muscle. In the case of predictive testing, we would advocate screening for the m.13094T>C mutation in muscle and/or urine to minimize the chance of false-negative results. The variable tissue segregation was further evident and exemplified by the variable mutation load in multiple tissues at autopsy (Patients 1.2 and 13). Intriguingly, the variable phenotypic threshold effect associated with the m.13094T>C mutation would support the assertion that *MT-ND5* synthesis is a rate limiting step for complex I activity (Sazanov, 2015), given the relatively low mutant load resulting in severe phenotypic expression (Blok et al., 2007). Although we do not have serial measurements of the m.13094T>C mutant load in blood for individual patients, our data suggest that mutant mtDNA heteroplasmy level may decline with age, and we would hypothesize that there is a negative selection of this mutant mtDNA in bone marrow over time, as demonstrated in other commoner mtDNA mutations such as m.3243A>G and m.13513G>A. However, we concede there is a possibility of ascertainment bias in the older individuals with lower mutant heteroplasmy levels.

Patients 1.2 and 8 had MELAS/LS overlapping syndrome whilst Patient 13 had LS only. Patient 1.2 predominantly manifested with a childhood onset CNS disorder, with no evidence of myopathy (on clinical examination), cardiomyopathy, renal impairment or hepatic disease. However, in Patient 1.2, the mutant heteroplasmy levels were much higher in skeletal muscle (88%), heart (75%), kidney (83%) and liver (83%) compared to CNS tissues (~67%). Only patient 8 manifested with stroke-like episodes and subsequently a brainstem crisis in her 30s, yet the mutant mtDNA heteroplasmy levels in CNS tissues (~52%) were lower than in Patient 1.2. The tissue specificity of phenotypic expression in this mutation is intriguing, and the possible underlying reasons require further elucidation yet support the observation that mutant heteroplasmy level alone does not fully explain clinical manifestation (Gorman et al., 2016).

In summary, the m.13094T>C mutation exhibits highly variable neurological manifestations and is frequently associated with high disease burden and early mortality. Cortico-thalamic-cerebellar involvement appears to be a frequent finding in these patients compared to other rare mtDNA mutations, and may serve as a radiological biomarker of m.13094T>C – related mitochondrial disease. Moreover, our findings would suggest m.13094T>C–related mitochondrial disease is perhaps not as rare as originally thought. We would suggest that consideration should be given to screening the whole mtDNA genome in clinically relevant tissues, prior to proceeding to whole exome sequencing, in those who have tested negative for the commonly recognized disease causing genes and mtDNA point mutations. Once again, the observed clinical heterogeneity, often apparent lack of maternal inheritance, normal histological and biochemical muscle biopsy findings, and variability in tissue segregation in these cases, highlights the diagnostic challenges of mitochondrial disease caused by rare mtDNA variants. We suggest, these findings support a better understanding of m.13094T>C –related syndromes and their inherent clinical trajectory and as such will serve to aid a more timely diagnosis, inform accurate genetic counseling, and facilitate tailored therapeutic interventions.

## Appendix A Supplementary data

Supplementary material Image 1
