# Supplementary material for: MT-ND5 Mutation Exhibits Highly Variable Neurological Manifestations at Low Mutant Load
Source: eBioMedicine. 2018 Feb 24;30:86–93. doi: 10.1016/j.ebiom.2018.02.010 (PMC5952215; doi:10.1016/j.ebiom.2018.02.010)

Supplemental data

Criteria to define specific mitochondrial syndromes:

Leigh syndrome (LS) is defined based on the following features: (1) progressive neurological disease with motor and intellectual developmental delay; (2) signs and symptoms of brainstem and/or basal ganglia disease; (3) raised lactate levels in blood and/or cerebrospinal fluid (CSF); and (4) one or more of the following: (a) characteristic radiological features of LS (symmetrical T2-hyperintense lesions in the basal ganglia and/or brainstem); (b) typical neuropathological changes at postmortem, or (c) typical neuropathology in a similarly affected sibling.[1,2]

The diagnosis of mitochondrial encephalomyopathy, lactic acidosis and stroke-like episodes (MELAS) syndrome was based on the criteria outlined by Hirano and colleagues.[3] Stroke-like episode refers to the subacute presentation of focal neurological deficit, often in the presence of focal seizures, and corresponding cortical and subcortical changes on the MRI head which are not confined to a single vascular territory.

Patients with Leber hereditary optic neuropathy (LHON) present with bilateral subacute loss of central vision and the time course is defined by an expanding dense scotoma and a period of worsening visual acuities until a nadir is reached. Other structural, metabolic, toxic, and inflammatory causes were excluded.[4]

Spinocerebellar ataxia (SCA) is characterized by cerebellar dysfunction with or without spinal cord/peripheral sensory involvement.[5]

Clinical vignettes

Family 1

Patient 1 initially presented with bilateral, subacute, painless visual failure (accompanied by delayed visual evoked potentials) at age 18 years. The patient developed focal onset epilepsy at age 27 and had an episode of status epilepticus eight years later. A cranial MRI performed during the admission of status epilepticus showed T2-hyperintensities within the right thalamus and right posterior parietal region. The patient was managed with phenobarbitone, lamotrigine and L-arginine. Despite aggressive management of seizures, the patient continued to have refractory focal motor seizures, neuropsychiatric symptoms, and progressive cognitive impairment and died at the age of 37 years.

Family pedigree review revealed that her son (Patient 1.1) died from Leigh disease at age 8 months. His clinical notes indicated that he became blind and deaf at four months. Her daughter (Patient 1.2) was found to have optic atrophy at age four years and developed progressive ataxia and refractory epilepsy at age ten years. The daughter's cranial MRI showed stroke-like lesions and extensive changes in the brainstem and thalami. The daughter died at age 14 years, and a post-mortem study was performed.

Family 2

Patient 2 was referred to the neurology clinic at the age of 24 years. The patient first developed a progressive ataxia disorder in childhood. Clinical signs included an ataxic gait, reduced lower limb reflexes and bilateral pes cavus. Serum lactate, creatine kinase, thyroid function test and serum Vitamin E level were normal, and anti-gliadin and anti-tissue transglutaminase antibodies were negative. Electrophysiological testing confirmed a sensory-motor axonal polyneuropathy. Genetic testing for hereditary cerebellar ataxias including SCA 1, 2, 3, 6, 7, dentatorubral pallidoluysian atrophy (DRPLA) and Friedreich ataxia (FA) was normal. The patient reported mild muscle weakness and cognitive difficulties during clinic review three years later. Cranial MRI showed a right medial thalamic lesion. A tibialis anterior muscle biopsy was performed to assist diagnostic evaluation. She developed a stroke-like episode characterized by occipital seizure status and right occipital lobe lesion with restricted diffusion at age 30 years.

Following the diagnosis of mitochondrial disease in Patient2, her mother (Patient 2.1) was reviewed in the national mitochondrial clinic. Patient 2.1 reported long-standing but mild clumsiness and reduced balance, and cerebellar signs were identified on examination. A maternal aunt (Patient 2.2) was examined at age 74 years and had evidence of essential tremor and hesitant tandem gait. A maternal cousin (Patient 2.3) died at 18 months, and a post-mortem examination confirmed LS. Another maternal cousin (Patient 2.4) had been investigated for subacute onset diplopia, bilateral optic atrophy and cerebellar ataxia at age 13 years. Imaging at that stage revealed a lesion in the right medulla, with prolonged right-sided latency on brainstem evoked potentials testing. He was re-examined at age 43 years following a genetic diagnosis being made in the pedigree index case (Patient 2). He now has features of a spinocerebellar ataxia disorder and can walk but only for limited distances; using a wheelchair for longer distances. The maternal grandmother of the index was reported to have a progressive neurodegenerative disorder characterized by cerebellar degeneration, recurrent strokes and refractory seizures and died at age 49 years.

Family 3

Patient 3 was induced at 36 weeks due to intrauterine growth restriction and weighed 2.4kg. He had feeding difficulties and developed an episode of apnoea several hours after birth. He was monitored on the special care baby unit but did not require mechanical ventilation. He exhibited failure to thrive following discharge home but initially reached typical developmental milestones. He subsequently displayed developmental regression and developed new onset strabismus, apnoeic episodes and lactic acidaemia. Cranial MRI revealed extensive T2 signal abnormalities in the thalami, brainstem (central grey matter of midbrain, middle cerebellar peduncles, and dorsal lateral medulla) and upper cervical cord. He was ventilator-dependent and died at six months of age. The patient's mother (Patient 3.1) was asymptomatic.

Family 4

Patient 4 was the first child of healthy, non-consanguineous parents. He was born by normal delivery at full term. He had mildly delayed motor milestones (crawling at 12 months and walking at 18 months of age) but language acquisition was normal. There were no subsequent

concerns until he developed ptosis and divergent strabismus with vertical gaze palsy aged 4 years.

Cranial MRI at the age of four years eight months showed abnormal signal bilaterally in the brainstem and cerebellar peduncles. Spectroscopy in the middle cerebellar peduncle showed normal N-acetylaspartate but a double lactate peak. The plasma lactate was normal, but the cerebrospinal fluid (CSF) lactate was raised at 3.0 mmol/L (normal range 1.0 – 2.5 mmol/L).

The ophthalmoplegia and ptosis lasted 18 months before completely resolving. At the age of 5.5 years, he developed progressive ataxia that prevented him from walking independently and pyramidal tract signs, including bilateral ankle clonus. Cognition remained normal. At five years eight months, he was admitted with increasing sleepiness and found to have type II respiratory failure. For three weeks, this responded to nocturnal non-invasive ventilation but the sleepiness recurred with headaches. He had hypercapnia (pCO_2 9 kPa), hypertension (highest reading 175/100 mmHg) and dysphagia with a weak cough. He was managed with atenolol, amlodipine and 24-hour non-invasive ventilation but continued to enjoy soft diet by mouth. A few days before his 6th birthday, he developed a cough, following which he became unrousable and died from an asystolic cardiac arrest.

Family 5

Patient 5 was a 14-year old girl born to non-consanguineous parents, who achieved developmental milestones within the normal range, but reported symptoms of mild exercise intolerance throughout childhood. The patient presented with focal seizures involving her left leg that evolved to bilateral tonic-clonic seizures. She reported a preceding history of subacute onset of numbness in both legs. There was no family history of any neurological disorders. Cranial and spine MRI revealed T2 signal abnormalities in the cerebral peduncle, right frontal lobe and the ventral part of the cervical cord between levels C2 to C6. CSF analysis showed no cells, normal protein and glucose levels and was negative for CSF-restricted oligoclonal bands and aquaporin 4 antibody. A presumptive diagnosis of acute disseminated encephalomyelitis was made, and the patient was treated initially with methylprednisolone followed by intravenous immunoglobulin, and recovery was documented in two months. The patient represented nine months later with an acute onset of profuse vomiting, right hemiparesis, right sided numbness and epileptic encephalopathy. Repeat

cranial MR showed new T2-signal changes in the medial parts of both thalami, patchy involvement of both frontal lobes (worse on the right), left side of the cerebellum and an enlarged lesion in the right cerebral peduncle. The previous cervical cord signal changes had resolved. Repeat CSF analysis was again normal. Various pharmacological agents were trialed, including lamotrigine, levetiracetam, oxcarbazepine and topiramate; but were ineffective in preventing refractory focal seizures. The patient subsequently developed an episode of severe aspiration pneumonia, and respiratory failure complicated by a mixed metabolic and respiratory acidosis (serum lactate 18 mmol/L); warranting mechanical ventilation. A diagnosis of mitochondrial disease at this stage was considered, and the patient underwent a muscle biopsy. In addition, the patient was treated with intravenous L-arginine 10g/day for a week, with minimal improvement observed. Multi-focal myoclonus, paresis of downgaze and horizontal nystagmus were clinical signs noted during the same admission. T2-MRI head showed bilateral midbrain and enlarging thalamic lesions. Three months later, the patient represented with a severe global headache, bilateral ptosis, hypotension (blood pressure 80/40 mmHg) and respiratory distress (respiratory rate 40/min). The patient was ventilated via tracheostomy for two months but died of status epilepticus at the age of 16 years.

Family 6

Patient 6 was born to non-consanguineous healthy parents. He was a reluctant feeder, and displayed mild growth retardation and developmental delay. At the age of two years and three months, he presented acutely with ataxia and new-onset strabismus following an upper respiratory tract infection. There were no documented seizures. On examination, he had a left abducens nerve palsy, restricted upgaze, bilateral ptosis, marked cerebellar ataxia and hyperreflexia. CSF analysis excluded an infective cause, and cranial MRI revealed diffuse, symmetrical T2 signal abnormalities extending from the thalami to the brainstem. He was treated with a course of methylprednisolone with a little improvement documented in the complex eye movement disorder, but ataxia continued to progress.

Four months later, he was characterized for emergency intubation because of episodes of unresponsiveness and apnoea. Loss of central respiratory drive was responsible for failure of weaning from mechanical ventilation. He was ventilated via a tracheostomy for eleven

months but died at the age of 3 years. His mother (Patient 6.1) was asymptomatic and subsequently gave birth to a healthy child.

Family 7

Patient 7 was noted to have left pale optic disc at the age of 7 years detected at routine examination. He was closely monitored, and his visual acuity and color vision remained stable for several years. At the age of 13 years, he was found to have bilateral optic atrophy without major changes in his vision. However, he subsequently presented with episodes of subacute retrobulbar optic neuritis affecting both eyes and that was unresponsive to intravenous methylprednisolone (1g/day for five days per episode). He is now 20 years old with significant bilateral optic atrophy. His visual acuity is 6/60 in the right eye and counting fingers at 1 meter only in the left eye. Serial cranial MRI throughout his clinical course was normal.

His younger brother (Patient 7.1) was found to have some pallor of the optic disc when examined at age six years, with normal cranial MRI and electroretinogram following family screening. At age ten years, visual acuity was 6/6 in the right eye and 6/36 in the left eye. Color vision was mildly reduced in the left eye. Fundus examination at this stage showed bilateral temporal pallor of the optic disc (more marked in the left eye). Patients 7 and 7.1 are now aged 20 years and 21 years respectively, and they have not exhibited, to date, other additional clinical features. The index case tested negative for mutations in the *OPA1* gene and the three common mtDNA mutations associated with LHON. Whole exome sequencing did not identify the underlying genetic defect (methods described elsewhere[6,7]) leading to full mitochondrial genome sequencing. Their mother (Patient 7.2) was reported to be fit and healthy.

Family 8

The clinical phenotype and neuropathological studies (cerebellum) of this patient (Patient 8) have briefly been described previously [8,9]. A 34-year old woman presented with subacute onset of headache, visual disturbance, vomiting and vertigo. The patient reported initial visual symptoms of intermittent red and yellow circles, in her left temporal visual field, progressing to a complete left homonymous hemianopia, on confrontational testing, over the

ensuing three days. These were associated with left eyelid twitching. Head MRI revealed T2-hyperintensities in the right occipital lobe, mid-brain and bilateral thalami. MR angiography was normal. A trans-oesophageal echocardiogram was performed and revealed a patent foramen ovale, and a diagnosis of cardiac-embolic posterior circulation stroke was initially made.

Two months later the patient reported episodic, left sided paraesthesia involving face, tongue, arm and leg. The patient then presented with initially a right focal motor seizure involving arm and leg that evolved to a brief bilateral convulsion, and was discharged home on carbamazepine. Her condition rapidly deteriorated with the development of unilateral ptosis, cerebellar ataxia, dysarthria, worsening multi-focal myoclonus and involuntary jerking of her jaw over the subsequent six months. Carbamazepine was substituted for sodium valproate and clonazepam. However, the patient represented to hospital with a further 10-day history of episodic positive visual phenomena (colored rings in front of both eyes associated with visual blurring), which were suggestive of occipital lobe seizures; in addition to increased agitation. Clinical examination revealed a right temporal visual field defect. The patient died unexpectedly two days after admission and a post-mortem examination was performed. Her mother (Patient 8.1) did not exhibit any signs or symptoms consistent with a diagnosis of mitochondrial disease.

Family 9

Patient 9 is a 34 year old woman who had a normal birth and normal cognitive and motor development during infancy and childhood. At school, she was good both intellectually and at sports and very active.

She presented aged 27 years with developed subacute sensory loss in the right lower limb that progressed over days and lasted for one month. She subsequently had recurrent attacks of neurological dysfunction, one per year on average, usually with subacute onset and lasting for days or weeks. Neurological symptoms have been different in each episode and have included sensory loss in her upper or lower limbs, gait difficulty, hemianopia, color vision problems, dysphasia and apraxia. On occasions, she would be unaware of the symptoms with possible confusion. Aged 31 years, during one of the attacks, she suffered two generalized tonic-clonic seizures and commenced antiepileptics (levetiracetam and lamotrigine) since which no further seizure activity has been recorded. She was later initiated

in L-Arginine. Full recovery between attacks was reported apart from a mild decline in cognition.
No specific triggers for the episodes were elicited. She experienced headaches, although none prior to or during an attack. Past medical and family history was unremarkable. Neurological examination aged 34 years was normal apart from mild dysphasia and reflex asymmetry.

Blood lactate was elevated (4.48 mmol/L, reference 0.5-2.2). CSF lactate was also raised. Brain MRIs showed sequential appearance of cortical / cortical-subcortical nonenhancing lesions (right occipital and left parietal, occipital, temporal and frontal) as well as multiple subependymal non-enhancing nodules (TSC1 and TSC2 genetic analysis negative). The whole-body PET-CT scan showed no evidence of malignancy. EEG showed left frontocentral and frontotemporal slowing and no epileptiform features.
Muscle biopsy showed increase in lipid droplets and abnormality of the internal architecture of mitochondria. There were no COX deficient fibers or ragged-red fibers. Mitochondrial respiratory chain enzyme complex I, II+III, and IV activities were within normal limits. Formal neuropsychometry revealed signs of executive dysfunction and reduced speed of information processing.

Family 10

Patient 10 presented at the age of 22 years with a seven week history of progressive ptosis, diplopia and confusion. Antecedent medical and family history was unremarkable. On admission he was encephalopathic and in type II respiratory failure. On admission, there was reduced upgaze, bilateral ptosis, bilateral internuclear ophthalmoplegia and downbeat nystagmus, and a mild facial diplegia. There were mild proximal muscle weakness and areflexia.

He was admitted to the medical high dependency unit and treated with BiPAP. Vital capacity was greater than 3 making a neuromuscular basis for the Type II respiratory failure unlikely. MRI brain which demonstrated florid symmetrical brainstem lesions. Echocardiogram confirmed left ventricular hypertrophy. Overnight oximetry showed up to 64 dips per hour of PO2 with a minimum dip to 84%. Early morning blood gas showed a pH of 7.4, PO2 9.2, PCO2 7.3 and bicarbonate of 34.1. He was therefore commenced on nocturnal non-invasive ventilation (NIV) with improvement in breathing.

Muscle biopsy showed mild variation in fibers size and occasional angular atrophic fibers but no pathological features to suggest mitochondrial disease. Respiratory chain enzyme analysis was normal.

Following discharge, a partial recovery was made with continuing diplopia, corrected using prisms, requirement of nocturnal nonv-invasive ventilation (NIV), poor concentration and fatigue.

Family 11

Patient 11 is a 31 year old man. He was born at 34 weeks, following a normal pregnancy, and required oxygen for first 48 hours of life and was mildly jaundiced. Early motor and cognitive development was normal. At the age of two years, he was admitted to hospital with vomiting, dehydration and ketoacidosis. The vomiting lasted, intermittently, for six months duration and was associated with multiple ear infections and fevers. Aged five years there was a possible reduction in visual acuity, and aged nine years he developed sudden onset diplopia. MRI brain confirmed basal ganglia lesions and CSF lactate was raised. Aged 12 years he developed hearing loss over one week which improved over one year. Aged 13 years mobility deteriorated and he was unable to walk for several months. MRI confirmed a lesion in the left lentiform nucleus. A trial of L-Dopa was commenced without effect and he recovered spontaneously. Aged 14 years he lost vision in both eyes over eight weeks and bilateral optic atrophy was detected. He was subsequently registered as blind. Vision subsequently improved to 6/60 right eye and 3/36 left eye. Aged 16 years he reported mental tasks requiring concentration caused significant fatigue but could play football for more than 30 minutes. However, by 18 years he had developed extreme physical fatigue, necessitating wheelchair use, and suffered from gastrointestinal symptoms, including abdominal pain and nausea with food, and weight loss. From the age of 20 years, he developed imbalance and dystonic posturing of the right hand, requiring botulinum toxin injections. The dystonia has since become generalized. Aged 22 years he required a percutaneous gastrostomy tube insertion for feeding and since an ITU admission with pneumonia, he has required a long term tracheostomy tube and nocturnal ventilation. Most recent examination (aged 29 years) demonstrated bilateral optic atrophy, left sided divergent strabismus, nystagmus, facial weakness and dysarthria. In limbs there was generalized dystonia and increased tone with mild proximal muscle weakness and normal reflexes. Plasma lactate was 1.46 mmol/L (reference 0.5-1.8mmol/L). CSF lactate was raised at 3.3mmol/L (reference 1-2.0 mmol/L).

Muscle biopsy was normal. Respiratory chain enzyme analysis showed borderline low complex IV.

Family 12

A male baby was born after 38 weeks 2 days gestation, uneventfully. Parents were nonconsanguineous. His growth and development have been normal until 13 months of age. Exotropia was found out at the age of 13 months. At one year four months, he was found out to have external ophthalmoplegia and blepharoptosis. His MRI showed bilateral symmetrical brain stem lesion on T2-weighted image, and he was diagnosed to have Leigh disease. His development retarded gradually. At four years four months, his DQ was 46 and pointed out to have autism spectrum disorder (ASD).

Family 13

This patient suffered from severe encephalomyopathy with respiratory failure and high lactate levels during infancy. Cranial MRI showed bilateral brainstem and basal ganglia lesions. He was bedridden without eye movement and underwent tracheostomy for home mechanical ventilation and tube feeding. He died at the age of 14 years because of acute gastroenteritis and dehydration. There was no family history of suggesting a similar disease.

Biochemical assays of mitochondrial respiratory chain activity on cultured lymphoblastoid cells in the patient showed mild isolated complex I deficiency. The heteroplasmic m.13094T>C mutation was detected in various organs of postmortem examination in this patient, and was undetectable in his mother’s and brother’s blood samples.

References

1. Rahman S, Blok RB, Dahl HHM, Danks DM, Kirby DM, Chow CW, Christodoulou J, Thorburn DR. Leigh syndrome: Clinical features and biochemical and DNA abnormalities. *Annals of neurology* 1996; **39**(3): 343-51.
2. Baertling F, Rodenburg RJ, Schaper J, Smeitink JA, Koopman WJH, Mayatepek E, Morava E, Distelmaier F. A guide to diagnosis and treatment of Leigh syndrome. *Journal of Neurology, Neurosurgery & Psychiatry* 2014; **85**(3): 257-65.
3. Hirano M, Ricci E, Koenigsberger R, Defendini R, Pavlakis SG, DeVivo DC, DiMauro S, Rowland LP. MELAS: an original case and clinical criteria for diagnosis. *Neuromusc Disord* 1992; **2**: 125 - 35.
4. Man PYW, Griffiths PG, Brown DT, Howell N, Turnbull DM, Chinnery PF. The Epidemiology of Leber Hereditary Optic Neuropathy in the North East of England. *The American Journal of Human Genetics* 2003; **72**(2): 333-9.
5. Bird TD. Hereditary Ataxia Overview. 1998 Oct 28 [Updated 2016 Mar 3]. In: Pagon RA, Adam MP, Ardinger HH, et al., editors. GeneReviews® [Internet]. Seattle (WA): University of Washington, Seattle; 1993-2016. 2016.
6. Neveling K, Feenstra I, Gilissen C, Hoefsloot LH, Kamsteeg EJ, Mensenkamp AR, Rodenburg RJ, Yntema HG, Spruijt L, Vermeer S, Rinne T, van Gassen KL, Bodmer D, Lugtenberg D, de Reuver R, Buijsman W, Derks RC, Wieskamp N, van den Heuvel B, Ligtenberg MJ, Kremer H, Koolen DA, van de Warrenburg BP, Cremers FP, Marcelis CL, Smeitink JA, Wortmann SB, van Zelst-Stams WA, Veltman JA, Brunner HG, Scheffer H, Nelen MR. A post-hoc comparison of the utility of sanger sequencing and exome sequencing for the diagnosis of heterogeneous diseases. *Human mutation* 2013; **34**(12): 1721-6.
7. Wortmann SB, Koolen DA, Smeitink JA, van den Heuvel L, Rodenburg RJ. Whole exome sequencing of suspected mitochondrial patients in clinical practice. *J Inherit Metab Dis* 2015; **38**(3): 437-43.
8. Lax NZ, Pienaar IS, Reeve AK, Hepplewhite PD, Jaros E, Taylor RW, Kalaria RN, Turnbull DM. Microangiopathy in the cerebellum of patients with mitochondrial DNA disease. *Brain : a journal of neurology* 2012; **135**(Pt 6): 1736-50.
9. Lax NZ, Hepplewhite PD, Reeve AK, Nesbitt V, McFarland R, Jaros E, Taylor RW, Turnbull DM. Cerebellar ataxia in patients with mitochondrial DNA disease: A molecular clinicopathological study. *Journal of Neuropathology and Experimental Neurology* 2012; **71**(2): 148-61.

Patient	Complex I	Complex II	Complex III	Complex IV	Complex I:II
1·1[a]	**0·054**<br>(0·104 – 0.268)	0·059<br>(0·040 – 0·204)	-	**0.009**<br>(0.014 – 0.034)	-
3[b]	0.094<br>(0.104 +/- 0.036)<br>nmols NADH oxidised.min^{-1}.unit citrate $synthase^{-1}$	0.138<br>(0.145 +/- 0.047)<br>nmols DCPIP reduce.min^{-1}.unit citrate $synthase^{-1}$	0.897<br>(0.554 +/- 0.345)<br>x 10^{-3} K.sec^{-1}.unit citrate $synthase^{-1}$	1.470<br>(1.124 +/- 0.511)<br>x 10^{-3} K.sec^{-1}.unit citrate $synthase^{-1}$	0.681<br>(0.52-0.95)
4[b]	0.062<br>(0.104 +/- 0.036)<br>nmols NADH oxidised.min^{-1}.unit citrate $synthase^{-1}$	0.115<br>(0.145 +/- 0.047)<br>nmols DCPIP reduce.min^{-1}.unit citrate $synthase^{-1}$	1.320<br>(0.554 +/- 0.345)<br>x 10^{-3} K.sec^{-1}.unit citrate $synthase^{-1}$	0.959<br>(1.124 +/- 0.511)<br>x 10^{-3} K.sec^{-1}.unit citrate $synthase^{-1}$	0.539<br>(0.52-0.95)
6[c]<br>(lowest control value)	**58** (94.17)<br>nmol/min/UCS	64 (56.14)<br>nmol/min/UCS	**851** (955.6)<br>nmol/min/UCS	343 (335.4)<br>nmol/min/UCS	0.91<br>(< 10th percentile)
8[b,d]	**0.065**<br>(0.104 +/- 0.036)<br>nmols NADH oxidised.min^{-1}.unit citrate $synthase^{-1}$	**0.045**<br>(0.145 +/- 0.047)<br>nmols DCPIP reduce.min^{-1}.unit citrate $synthase^{-1}$	-	2.780<br>(1.124 +/- 0.511)<br>x 10^{-3} K.sec^{-1}.unit citrate $synthase^{-1}$	-
9[a]	0.19<br>(0.104 – 0.268)	0.071<br>(0.04 – 0.204)	-	0.016<br>(0.014 – 0.034)	Normal
10[a]	0.147<br>(0.104 – 0.268)	0.121<br>(0.04 – 0.204	-	0.015<br>(0.014 – 0.034)	-
11[a]	Normal	Normal	Normal	Low normal	-
12[e]	**199**<br>(222-473)<br>mU/U CS	243<br>(209-425)<br>mU/U CS	261<br>(53-296)<br>mU/U CS	29<br>(14-62)<br>mU/U CS	818<br>(599-1345)<br>(mU/U CII)
13[e]	**9**<br>(222-473)<br>mU/U CS	355<br>(209-425)<br>mU/U CS	101<br>(53-296)<br>mU/U CS	**4**<br>(14-62)<br>mU/U CS	**26**<br>(599-1345<br>(mU/U CII)

Supplemental Table 1. Measurement of mitochondrial respiratory chain activity in skeletal muscle. [a]Muscle samples of Patients 1.1, 9, 10 and 11 were analysed using the methods described by Oppenheim et al. 2007; [b]muscle samples of Patients 3, 4 and 8 were analysed based on the methods described by Taylor et al. 2004; [c]Two criteria is used to identify the presence of a respiratory chain enzyme deficiency: firstly, the activity value should be lower than the lowest control value when expressed on CS; secondly the activity also have to be less than at least the 5th percentile of the control group when expressed against either CII or CIV, if not deficient (if both are deficient only the first criterion is applied). This muscle biopsy showed evidence of complex I deficiency deficient as its activity was lower than lowest control value expressed on CS, lower than the 10th percentile of the reference range when expressed on CII and also lower than the 5th percentile of the reference range when expressed on CIV (not shown). The low complex III activity was likely secondary to the complex I deficiency. Methods described elsewhere by Smuts et al. 2010; [d]Whilst the complex IV activity appeared normal, the abnormal results of complex I and II could be caused by the significant delay in handling of the post mortem muscle tissue. [e]Citrate synthase activity was within normal limit, methods described by Kirby et al. 2007. CS= citrate synthase, DCPIP= dichlorophenolindophenol, NADH= Nicotinamide adenine dinucleotide

We have not had the original report on the biochemical analysis for patient 2.4 (performed in the early 90s) but the patient was documented to have normal respiratory chain activity analysis in the muscle biopsy

References

1. Oppenheim MLS, Hargreaves IP, Pope S, Land JM, Heales SJR. Mitochondrial cytochrome c release: a factor to consider in mitochondrial disease? *Journal of Inherited Metabolic Disease* 2009; **32**(2): 269-73.

2. Taylor RW, Schaefer AM, Barron MJ, McFarland R, Turnbull DM. The diagnosis of mitochondrial muscle disease. *Neuromuscular disorders : NMD* 2004; **14**(4): 237-45.

3. Smuts I, Louw R, Toit H, Klopper B, Mienie L, Westhuizen F. An overview of a cohort of South African patients with mitochondrial disorders. *Journal of Inherited Metabolic Disease* 2010; **33**(3): 95-104.

4. Kirby DM, Thorburn DR, Turnbull DM, Taylor RW. Biochemical assays of respiratory chain complex activity. *Methods in cell biology* 2007; **80**: 93-119.

Supplemental Table 2 – Summary of the macro- and microscopic neuropathological findings of two patients harbouring the m.13094T>C mutation.

	Patient 1.2	Patient 8
Frontal lobe	Neuronal population density in the pre-frontal cortex is intact.	Posterior frontal cortex reveals two foci of abnormal cortex with severe microvacuolation, capillary proliferation, severe neuronal cell loss, and astrogliosis, though morphologically normal neurons are present within the area of the lesion.
Temporal lobe and hippocampus	Temporal lobe is not available for analysis. The hippocampus demonstrates normal neuronal population density.	The temporal cortex reveals a minor degree of disruption, neuronal cell loss and capillary proliferation and mild to moderate vacuolation of underlying white matter. There is severe microvacuolation of the stratum lacunosum moleculare extending for most of the length of dentate gryus.
Occipital lobe	The neuronal layers within the occipital cortex appeared entirely preserved however there were spongiform changes occurring in the deep white matter.	Multiple lesions are evident in the primary visual cortex including capillary proliferation, microvacuolation, severe neuronal cell loss (affecting cortical layers I, III, V and VI) and astrogliosis. A partial old infarct (2x4 mm) is present in white matter underlying the primary visual cortex.
Basal ganglia	Tissues not available.	The caudate and putamen demonstrate intact neuronal population density. The mammillary body shows focal neuronal degeneration and foci of neurons with shrunken nuclei. Minor lesions evident in the hypothalamus.
Thalamus and subthalamus	Tissues not available.	The pulvinar and subthalamic nuclei are devastated showing prominent capillary proliferation, microvacuolation and severe neuronal cell loss and astrogliosis. There are morphologically well-preserved neurons scattered throughout.
Midbrain	The midbrain demonstrates severe cavitation with microvacuolation, severe neuronal cell loss and severe neuronal cell dropout.	Devastating lesion affecting the lateral border of periaqueductal grey, cuneiform, peduculo-pontine and inferior collicular nuclei with prominent arteriolar and capillary proliferation, microvacuolation and severe neuronal cell loss.
Pons and medulla	Macroscopically the basis pontis was affected by a devastating lesion which resulted in severe microvacuolation and total neuronal cell loss. The inferior olives demonstrated a profound loss of neurons without any evidence of lesion.	Lesion consisting of capillary proliferation and microvacuolation at the ponto-medullary junction affecting the superior raphe nucleus. Focal cell loss affecting the inferior olivary nucleus.
Cerebellum	The posterior cerebellar cortex demonstrated multiple areas of necrotic lesions ranging from atrophy of the molecular layer, Purkinje cell dropout and granule cell loss to total necrosis of the cerebellar cortex and also affecting the underlying white matter. In non-lesioned areas of the cerebellum, Purkinje cell density was reduced. The cerebellar deep white matter showed profound demyelination which may be related to the necrotic changes in the cortex.	The cerebellum was more preserved despite evidence of “staghorn” hypertrophy of secondary and tertiary dendrites in numerous Purkinje cells. The dentate nucleus showed focal neuronal cell loss, up to 50% in total.

Supplemental Figure Legend

Supplemental Figure 1. Family pedigrees and m.13094T>C heteroplasmy level in different tissues. Family trees are not available for Patients 9, 10 and 11. ⊙= carrier, B= blood, Bu= buccal mucosa, CVB= chorionic villous sampling, F= fibroblast, H= hair follicle, LS= Leigh syndrome, LHON= Leber hereditary optic neuropathy, M= muscle, MELAS= mitochondrial encephalomyopathy, lactic acidosis and stroke-like episodes, n.d.= not detectable, SCA= spinocerebellar ataxia, U= urinary epithelial cells.

Supplemental Figure 2. Serial T2-weighted axial MRI of Patient 12 (a to l). At 1 year 4 months, there were bilateral symmetrical high T2 signal intensiteis (with restricted diffusion) in the medulla oblongata, pontine tegmentum, around the aqueduct of the midbrain, subthalamic nucleus and anterior of thalamus.(a,b,c). Over a 6 month interval (age 16 months to 22 months; d,e,f). These signal abnormalities showed partial resolution in subsequent MRI head scans performed at aged 2 years 4 month (g,h,i) and 3 years 8 months (j,k,l), respectively.

Supplemental Figure 3: Downregulation of complex I subunit relative to high mitochondrial mass in CNS tissues.

Patient 1.2: The pontine nucleus neurons have variable complex I subunit expression with some neurons demonstrating an absence of this protein (A; NDUFB8 IHC), while mitochondrial mass is relatively high (B; SDHA IHC). The affected cerebellar cortex reveals a loss of complex I subunit expression (C; NDUFB8 IHC) while mitochondrial density is retained in the small number of surviving cells (D; SDHA

IHC). Occipital cortex neurons demonstrate variable complex I subunit expression (E; NDUFB8 IHC) despite high mitochondrial mass (F; SDHA IHC). Scale bar = 100 microns.

Patient 8: There are no complex I positive cells remaining in the lesioned thalamus (G; NDUFB8 IHC) while mitochondrial density is maintained (H; SDHA IHC). The lesioned occipital cortex reveals low abundance of complex I in remaining neurons (I; NDUFB8 IHC) while retaining high mitochondrial mass (J; SDHA IHC). In normal appearing occipital cortex, neurons show variable expression of complex I subunit (K; NDUFB8 IHC) while mitochondrial mass is uniformly high (L; SDHA IHC).

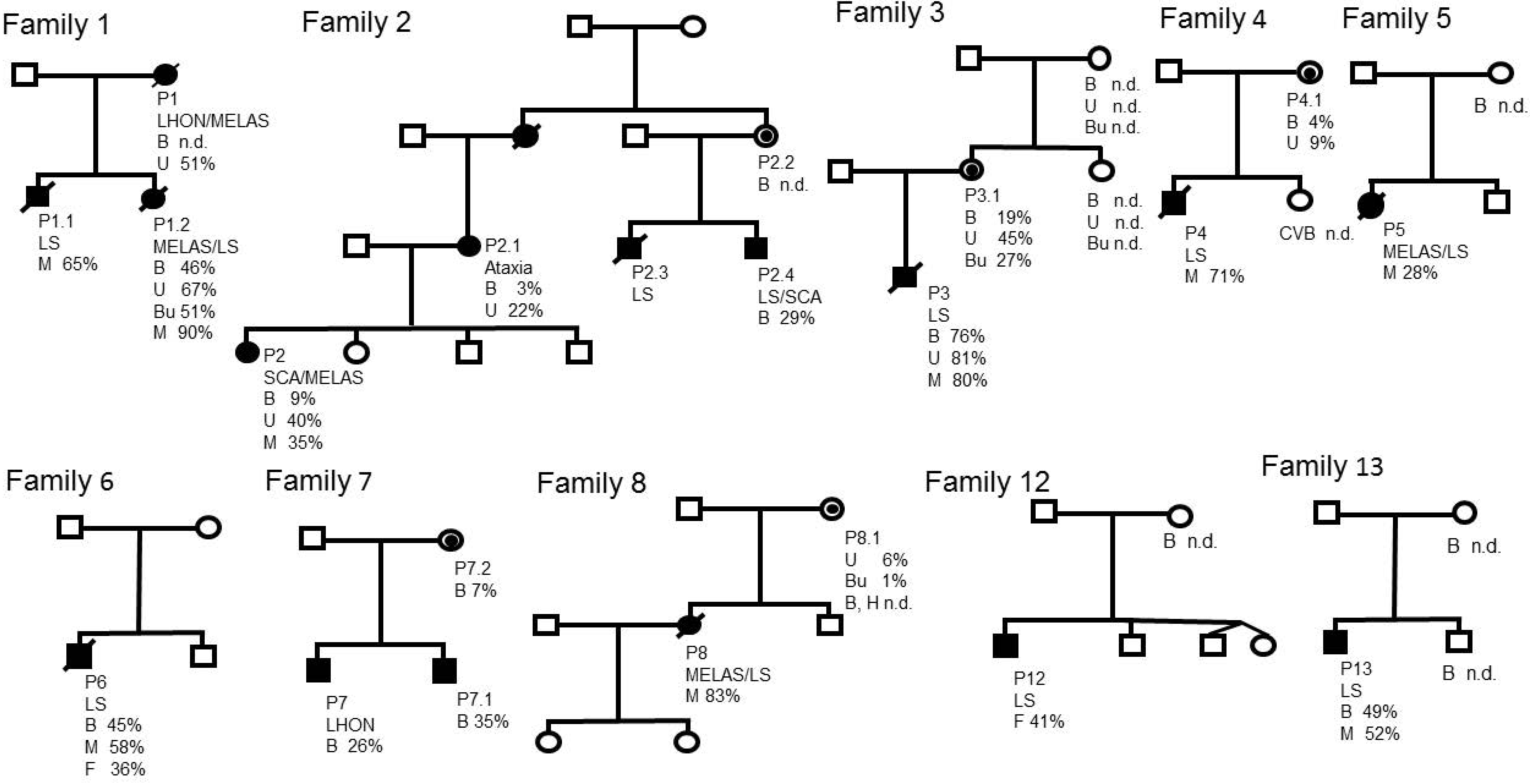
Family 1
P1
LHON/MELAS
B n.d.
U 51%
P1.1
LS
M 65%
P1.2
MELAS/LS
B 46%
U 67%
Bu 51%
M 90%
Family 2
P2.1
Ataxia
B 3%
U 22%
P2.2
B n.d.
P2.3
LS
P2.4
LS/SCA
B 29%
P2
SCA/MELAS
B 9%
U 40%
M 35%
Family 3
B n.d.
U n.d.
Bu n.d.
P3.1
B 19%
U 45%
Bu 27%
B n.d.
U n.d.
Bu n.d.
P3
LS
B 76%
U 81%
M 80%
Family 4
P4.1
B 4%
U 9%
P4
LS
M 71%
CVB n.d.
Family 5
B n.d.
P5
MELAS/LS
M 28%
Family 6
P6
LS
B 45%
M 58%
F 36%
Family 7
P7.2
B 7%
P7
LHON
B 26%
P7.1
B 35%
Family 8
P8.1
U 6%
Bu 1%
B, H n.d.
P8
MELAS/LS
M 83%
Family 12
B n.d.
P12
LS
F 41%
Family 13
B n.d.
P13
LS
B 49%
M 52%
B n.d.

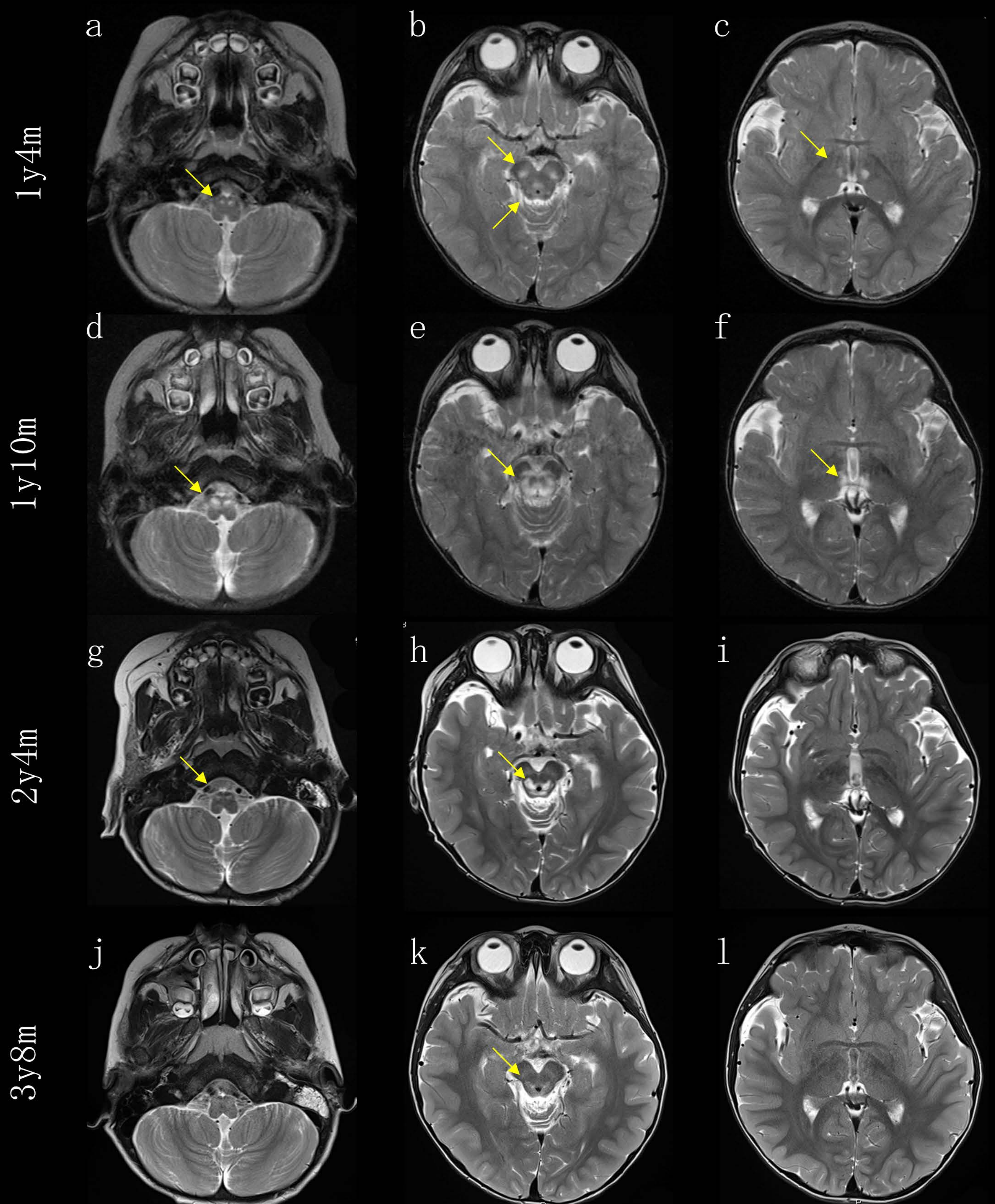
a
b
c
d
e
f
g
h
i
j
k
l
1y4m
1y10m
2y4m
3y8m

Supplemental Figure 3

Patient 1.2

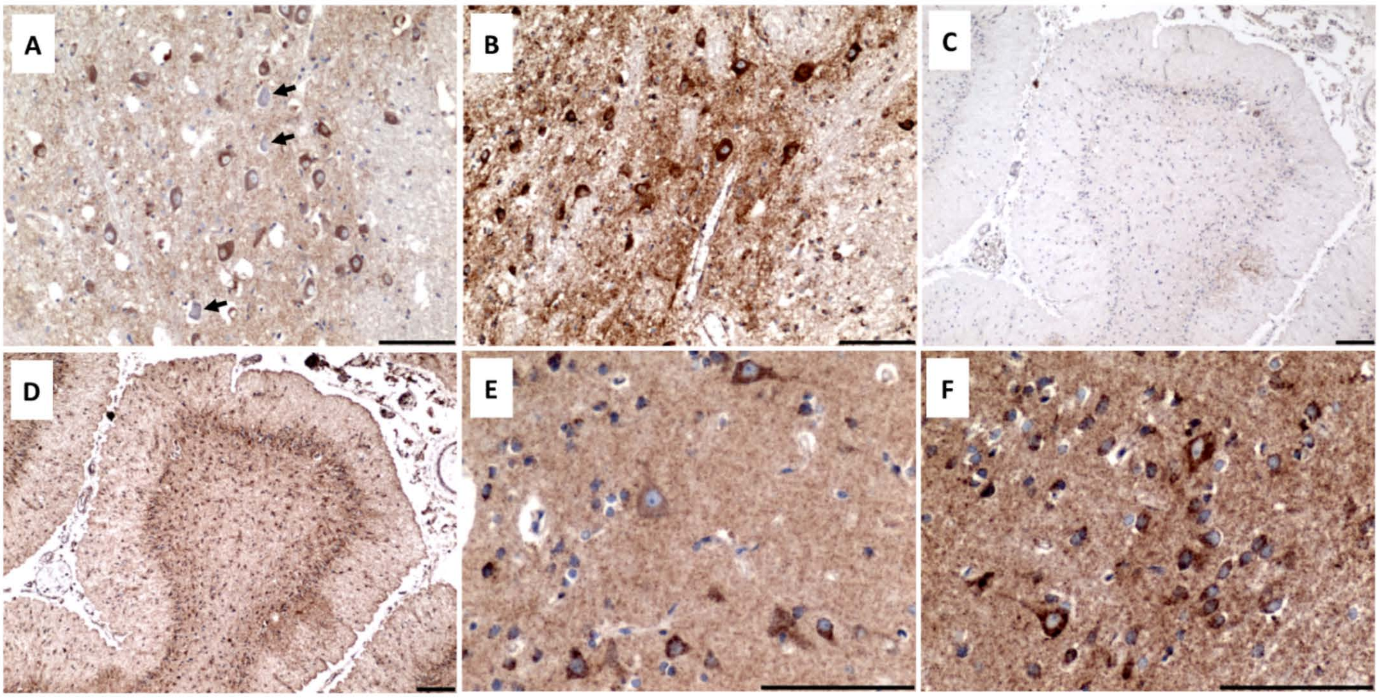

Patient 8

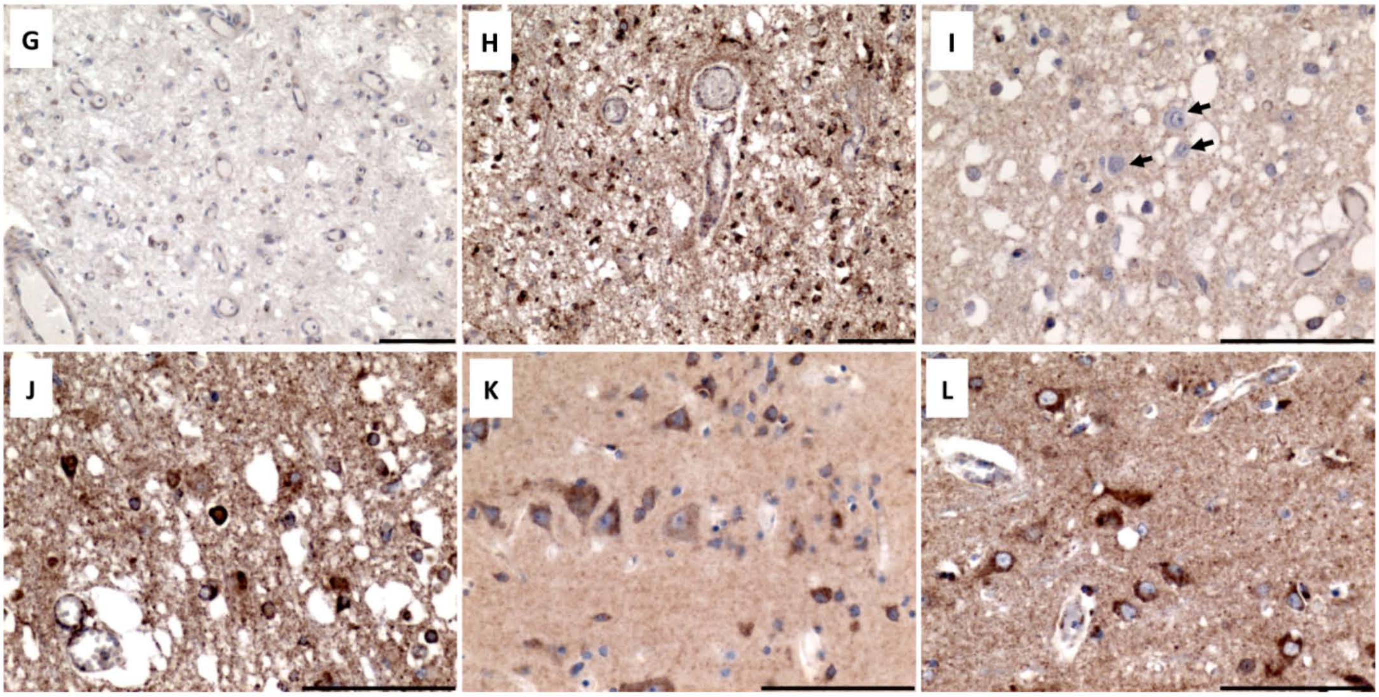

Supplement: Supplementary file 1 — Supplementary material [file mmc1.pdf]
